# Comparable Outcomes Without Routine Pan-mucosal Irradiation in Head and Neck Carcinoma of Unknown Primary

**DOI:** 10.1016/j.adro.2026.102130

**Published:** 2026-07-03

**Authors:** Mahmoud Allaf, Linda Diederich, André Buchali, Heidi Olze, Carmen Stromberger, Achim Franzen, Annekatrin Coordes

**Affiliations:** a Department of Otorhinolaryngology, Head and Neck Surgery, University Medical Centre Ruppin Brandenburg (ukrb), Brandenburg Medical School, Neuruppin, Germany; b Faculty of Health Sciences Brandenburg, Joint Faculty of the University of Potsdam, Brandenburg University of Technology Cottbus-Senftenberg and Brandenburg Medical School, Potsdam, Germany; c Charité – Universitätsmedizin Berlin, corporate member of Freie Universität Berlin, Humboldt-Universität zu Berlin, and Berlin Institute of Health, Department of Otorhinolaryngology, Head and Neck Surgery, Campus Benjamin Franklin, Berlin, Germany; d Department of Radiooncology, University Medical Centre Ruppin Brandenburg (ukrb), Brandenburg Medical School, Neuruppin, Germany; e Charité – Universitätsmedizin Berlin, corporate member of Freie Universität Berlin, Humboldt-Universität zu Berlin, and Berlin Institute of Health, Department of Otorhinolaryngology, Head and Neck Surgery, Campus Virchow Klinikum and Campus Charité Mitte, Berlin, Germany; f Charité – Universitätsmedizin Berlin, corporate member of Freie Universität Berlin, Humboldt-Universität zu Berlin, and Berlin Institute of Health, Department of Radiooncology, Campus Virchow Klinikum, Berlin, Germany

## Abstract

**Purpose:**

Evaluate whether adding pan-mucosal irradiation (50 Gy) reduces recurrence or improves survival in head and neck carcinoma of unknown primary.

**Methods and Materials:**

Retrospective 2-center analysis (center 1, 2009-2018, and center 2, 2005-2023). All patients underwent neck dissection with adjuvant irradiation to lymphatic drainage or definitive radiochemotherapy; center 2 additionally received 50 Gy pan-mucosal irradiation to the upper aerodigestive tract. Definitive radiation therapy/chemoradiation therapy was used for advanced nodal disease.

**Results:**

Of 164 screened, 134 were included (center 1, n = 88, and center 2, n = 46). Median follow-up was 72 months (95% CI, 48-96). Sixty-five patients (42%) died (37 vs 28). Overall survival did not differ between centers (log-rank *P* = .899). Combining centers 1 and 2, there were 39 recurrences in 134 patients; 24 out of 88 patients (27.3%) in center 1 and 15 out of 46 patients in center 2 (32.6%), *P* = .519. There were no differences noted in time-to-event analysis (log-rank *P* = .724). Mucosal recurrences occurred in 13 patients (9 vs 4; *P* = .282); all arose more than 36 months after diagnosis and exclusively in persistent smokers.

**Conclusion:**

In thoroughly staged head and neck carcinoma of unknown primary, adding pan-mucosal irradiation to standard nodal irradiation was not associated with improved survival or lower mucosal recurrence rates compared with nodal-only fields. The late occurrence of mucosal events and their association with persistent smoking suggest second primary tumors rather than missed index lesions. Given potential toxicity and quality-of-life trade-offs, routine pan-mucosal irradiation may not be warranted, and treatment should be individualized.

## Introduction

Head and neck cancer unknown primary (HNCUP) refers to patients presenting with cervical lymph node metastases without an identifiable primary tumor. Histologically, most cases are undifferentiated carcinoma and head and neck squamous cell carcinoma (HNSCC), accounting for approximately 90%.^1^, ^2^, ^3^ HNCUP represents a minority within HNSCC, comprising about 1% to 5% of cases.^1^^,^^4^, ^5^, ^6^ Established risk factors include human papillomavirus (HPV) infection (particularly HPV-16),^7^^,^^8^ tobacco use, heavy alcohol consumption, and Epstein-Barr virus (EBV) infection. Etiologically, HPV is associated with oropharyngeal squamous cell carcinoma (OPSCC),^9^ whereas EBV is linked to nasopharyngeal carcinoma.^9^, ^10^, ^11^ Classification of HNCUP is informed using surrogate viral markers analogous to oropharyngeal squamous cell carcinoma and nasopharyngeal carcinoma, specifically p16 (for HPV) and EBV status.^12^, ^13^, ^14^, ^15^ Additional staging considerations include the number and size of involved lymph nodes, laterality (ipsilateral, contralateral, or bilateral), and—among p16- and EBV-negative HNCUP—extranodal extension (ENE).^14^

A diagnostic work-up typically entails panendoscopy with directed biopsies or mucosectomy of the tongue base and unilateral or bilateral tonsillectomy.^16^ Contrast-enhanced computed tomography (CT) and positron emission tomography (PET)/CT scans are used to identify occult primaries.^17^ Treatment strategies include neck dissection (ND) followed by adjuvant radio(chemo)therapy or definitive radio(chemo)therapy.^18^, ^19^, ^20^ Prophylactic irradiation of at-risk mucosal sites (50-66 Gy in 2.0 Gy fractions) aims to prevent the emergence of a mucosal primary in the head and neck region.^18^ Indications for mucosal irradiation remain heterogeneous across institutions.^21^

The aim of the study was to examine whether mucosal irradiation prevents recurrence as part of the therapy for patients with HNSCC.

## Methods and Materials

### Study design and setting

We conducted a retrospective, multicenter cohort study of patients treated for HNCUP at 2 institutions: center 1 (2009-2018)^22^ and center 2 (2005-2023). The study was approved by both respective institutional review boards. Medical records of all patients with HNCUP were reviewed, including medical history, imaging, operative reports, and histopathology.

### Patient inclusion and data collection

Eligible patients had cervical lymph node metastases without an identifiable primary tumor after standard diagnostic work-up. For all included patients, we abstracted demographic, clinical, imaging, surgical, pathological, and treatment data from electronic records.

### Diagnostic assessment

Diagnostic assessment of all patients with HNCUP included medical history, physical examination, and radiological imaging studies (CT scan or magnetic resonance imaging of the neck and CT scan of the thorax and abdomen). A PET-CT scan was performed in 53 patients in center 1. Each patient in both centers underwent panendoscopy for primary tumor search; biopsies were taken from the nasopharynx and tongue base, and tonsillectomies were performed bilaterally. The lymph node was removed or biopsied for histopathologic evaluation. The diagnoses were histologically confirmed in all cases by pathologic institutions. The Union for International Cancer Control (UICC) stage was classified according to the TNM Classification of Malignant Tumours (TNM), seventh and eighth editions.

### Multidisciplinary treatment planning

All cases were discussed in a multidisciplinary tumor board including head and neck surgeons, radiation oncologists, pathologists, medical oncologists, and radiologists. Treatment recommendations were based on the UICC stage and the patient’s clinical condition, in accordance with the National Comprehensive Cancer Network guidelines.^18^

### Surgical and radiation therapy management

For patients managed with upfront surgery, ND was performed first. Depending on suspected infiltration of adjacent structures, ND was carried out as a modified radical or radical procedure. Laterality (unilateral vs bilateral) was determined based on clinical and radiological suspicion of contralateral nodal involvement. Postoperative treatment consisted of adjuvant radiation therapy (RT) or chemoradiation therapy (CRT). Patients who did not undergo ND received definitive RT with or without concurrent chemotherapy.

RT dose prescriptions were based on nodal disease extent and the presence of ENE. In the postoperative setting, patients with pathologically involved lymph nodes without ENE received 50 to 56 Gy, whereas patients with ENE received 64 to 66 Gy. In the definitive setting, patients with macroscopic nodal disease received >70 Gy to gross tumor volumes, although elective doses were delivered to regional nodal areas according to institutional standards.

RT techniques evolved over time in both centers. In center 1, intensity-modulated RT (IMRT) techniques were used throughout the study period, with a transition to volumetric modulated arc therapy from 2011 onward, typically using 2 to 3 arcs or partial arcs depending on target volume definition. All treatments were delivered using Varian Medical Systems linear accelerators. Treatment planning aimed to ensure adequate target coverage while sparing organs at risk, including the parotid glands and spinal cord, according to institutional standards. Adjuvant irradiation in center 1 was directed to the involved neck and elective lymphatic drainage following ND, whereas the upper aerodigestive tract mucosa was not included in the elective clinical target volume.

In center 2, adjuvant lymphatic irradiation was combined with pan-mucosal irradiation of the upper aerodigestive tract to a dose of 50 Gy. In this study, pan-mucosal irradiation was defined as elective irradiation of potential occult primary mucosal sites, including the nasopharynx, oropharynx, hypopharynx, and larynx. Irradiation of the oral cavity mucosa was additionally performed in patients with cervical lymph node metastases involving levels I–II. Definitive RT or CRT was used in patients who did not undergo ND and in selected patients with advanced nodal disease. Concurrent CRT was administered postoperatively in the presence of ENE and definitively in patients with macroscopic nodal metastases. Systemic therapy consisted of cisplatin with or without 5-fluorouracil or mitomycin C with or without 5-fluorouracil, according to institutional standards and treatment era.^17^

Patients with advanced UICC stage disease presenting with distant metastases and/or poor performance status received palliative systemic therapy (eg, cetuximab or nivolumab) and best supportive care.^17^ These patients were not included in the present study.

### Immunohistochemistry and in situ hybridization

Immunohistochemical staining for p16 was performed on a BenchMark ULTRA autostainer (Ventana) using a monoclonal rabbit antibody (p16INK4a; CINtec Histology Kit; Ventana Medical Systems, Inc). p16 overexpression—used as a surrogate for HPV-related disease—was defined as moderate to strong (2+/3+) nuclear staining in ≥75% of tumor cells.^23^ EBV in situ hybridization was performed on a Leica BOND-MAX stainer (Leica Biosystems, IL, USA) using a fluorescein-conjugated oligonucleotide probe according to manufacturers’ instructions.

### Outcomes

Clinicopathologic variables evaluated included sex (male vs female), age (<65 vs ≥65 years), tobacco exposure (never vs ever), alcohol exposure (yes vs no), history of additional cancers (yes vs no), p16 status (positive vs negative), EBV status (positive vs negative), ENE (yes vs no), N classification (UICC 7 and UICC 8), and UICC stage (TNM 7 and TNM 8).

The primary outcome was overall survival (OS), defined as the time from initial HNCUP diagnosis to death from any cause or last follow-up.

Disease-free survival (DFS) was defined as the time from initial HNCUP diagnosis to tumor recurrence at any location with detectable squamous cell carcinoma or last follow-up.

Separately, we evaluated the tumor recurrence of the mucosa of the upper aerodigestive tract. This can be discussed as the primary tumor location of the HNCUP.

### Statistical analysis

Continuous variables are reported as mean ± standard deviation following the Statistical Analyses and Methods in the Published Literature reporting guidelines.^24^ Categorical variables are presented as counts and percentages. Group comparisons of continuous variables used analysis of variance; associations between categorical variables used Fisher’s exact test.

OS and DFS were estimated using the Kaplan–Meier method. All *P*-values are 2-sided and considered statistically significant at *P* < .05. Analyses are exploratory; no adjustment for multiple testing was performed. Statistical analyses were conducted using IBM SPSS Statistics, version 25.2 (IBM Corp).

## Results

### Patient characteristics and treatment

At center 1, 110 patients were diagnosed with HNCUP and histologically confirmed HNSCC between 2009 and 2018; results for 97 of these patients have been reported previously.^22^ At center 2, 54 patients were diagnosed between 2005 and 2023. All patients included in this analysis completed the therapy recommended by the multidisciplinary tumor board: 88 of the 110 (80.0%) in center 1 and 46 of the 54 (85.2%) in center 2.

Clinical and pathologic characteristics are summarized in Table 1. The mean age at HNCUP diagnosis was higher in center 1 than in center 2 (64.7 vs 56.5 years; *P* < .001). The proportion of male patients was 70.5% in center 1 and 91.3% in center 2 (*P* = .006). Overall, 73.6% were current smokers (78.5% in center 1 vs 66.7% in center 2; *P* = .167). A history of prior malignant disease was present in 14.4% (center 1) and 11.1% (center 2), including breast, kidney, lung, and colorectal cancers (Table 2).

**Table 1:** Patient and tumor characteristics of the study population

Variable n	Total N = 134	Center 1 N = 88	Center 2 N = 46	*P*-value
Patient characteristics				
Sex, n (%)				.006
Female	30 (22.4)	26 (29.5)	4 (8.7)	
Male	104 (77.6)	62 (70.5)	42 (91.3)	
Age at initial diagnosis of HNSCC, y				<.001*
Mean (±SD)	61.6 (11.2)	64.4 (10.9)	56.2 (9.6)	
Smoking habits, n (%)				.167
Smoker	81 (73.6)	51 (78.5)	30 (66.7)	
Nonsmoker	29 (26.4)	14 (21.5)	15 (33.3)	
Alcohol abuse, n (%)				.295
None	95 (70.9)	65 (73.9)	30 (65.2)	
Yes	39 (29.1)	23 (26.1)	16 (34.8)	
HNSCC characteristics				
P16				.136
Positive	32 (25.0)	24 (29.3)	8 (17.4)	
Negative	96 (75.0)	58 (70.7)	38 (82.6)	
EBV				1.000‡
Positive	3 (3.6)	3 (3.7)	2 (4.3)	
Negative	79 (96.4)	99 (96.3)	44 (95.7)	
ECS				.512
Positive	78 (58.2)	53 (60.2)	25 (54.3)	
Negative	56 (41.8)	35 (39.8)	21 (45.7)	
Grading, n (%)				.014†
G1	1 (0.9)		1 (2.2)	
G2	52 (45.2)	40 (45.5)	12 (26.1)	
G3	62 (53.9)	33 (37.5)	29 (63.0)	
N classification (UICC 7), n (%)				.088
1	18 (13.4)	12 (13.6)	6 (13.0)	
2a	31 (23.1)	25 (28.4)	6 (13.0)	
2b	62 (46.3)	39 (44.3)	23 (50.0)	
2c	5 (3.7)	1 (1.1)	4 (8.7)	
3	18 (13.4)	11 (12.5)	7 (15.2)	
N classification (UICC 8), n (%)				.019
1	36 (26.9)	26 (29.5)	10 (21.7)	
1a	1 (0.7)	0	1 (2.2)	
2	3 (2.2)	2 (2.3)	1 (2.2)	
2a	24 (17.9)	19 (21.6)	5 (10.9)	
2b	18 (13.4)	15 (17.0)	3 (6.1)	
2c	2 (1.5)	1 (1.1)	1 (2.2)	
3	3 (2.2)	3 (3.4)	0	
3a	2 (1.5)	0	2 (4.3)	
3b	45 (33.6)	22 (25.0)	23 (50.0)	
UICC 7 stage, n (%)				.328
3	23 (17.2)	18 (20.5)	5 (10.9)	
4a	89 (66.4)	58 (65.9)	31 (67.4)	
4b	17 (12.5)	10 (11.4)	7 (15.2)	
4c	5 (3.7)	2 (2.3)	3 (6.5)	
UICC 8 stage, n (%)				.175
1	27 (20.1)	20 (22.7)	7 (15.2)	
2	3 (2.2)	2 (2.3)	1 (2.1)	
2a	1 (0.7)	1 (1.1)	0	
3	9 (6.7)	6 (6.8)	3 (6.5)	
4a	41 (30.6)	30 (34.0)	11 (23.9)	
4b	49 (36.6)	28 (31.8)	21 (45.7)	
4c	3 (2.2)	0	3 (6.5)	

*Abbreviations*: EBV = Epstein-Barr virus; ECS = extracapsular spread; HNSCC = head and neck squamous cell carcinoma; UICC = Union for International Cancer Control.

⁎ t-test; Levene Test 0.138.

^†^ The requirements to perform a *χ^2^* test were not fulfilled.

^‡^ Fisher’s exact test*.*

**Table 2:** Additional primary cancers

Localisation	Total N = 25	Center 1 N=17	Center 2	p-Value = 0.310
Intestinal Cancer	4 (84.3)	2 (83.0)	2 (87.0)	
Skin Cancer	2 (85.8)	2 (85.2)	0	
Bronchial Cancer	4 (88.8)	2 (87.5)	2 (91.3)	
Lymphoma	1 (89.6)	0	1 (93.5)	
MammaCA	6 (94.0)	5 (93.2)	1 (95.7)	
Kidney Cancer	2 (95.5)	0	2 (100.0)	
ÖsophagusCA	1 (96.3)	1 (94.3)	0	
PenisCA	1 (97.0)	1 (95.5)	0	
ProstataCA	3 (99.3)	3 (98.9)	0	
ThyroidCA	1 (100.0)	1 (100.0)	0	

ND (Table 3) was performed in 57.0% of patients in center 1 and 91.0% in center 2. Bilateral ND was undertaken in 2.3% (center 1) and 23.1% (center 2). Overall, 26.9% of patients received definitive RT or CRT.

**Table 3:** Therapy of the HNCUP patients

Localisation	Total N=134	Center 1 N=88	Center 2 N=46
Neck dissection	98 (73.1)	56 (63.6)	42 (91.3)
ipsilateral	83 (61.9)	52 (59.1)	31 (67.4)
bilateral	13 (9.7)	2 (2.3)	11 (23.4)
and parotidectomy	7 (5.2)	2 (2.3)	5 (10.9)
Adjuvant radiotherapy	39 (29.1)	23 (26.1)	16 (34.8)
Adjuvant RCT	56 (41.8)	32 (36.4)	24 (52.2)
No adjuvant therapy	2 (1.5)	0	2 (4.8)
Definitive RCT	32 (23.9)	28 (31.8)	4 (8.7)
Definitive RT	4 (3.0)	4 (4.5)	0 (0.0)

Abbreviation: RCT, radiochemotherapy; RT, radiotherapy.

### Tumor characteristics

All tumors were histologically confirmed as HNSCC. Extranodal extension spread was observed in 58.2% overall (60.2% in center 1; 54.6% in center 2). According to the UICC TNM seventh and eighth editions, most patients presented with advanced nodal categories and stages at initial diagnosis (Table 1). Distant metastases at presentation were identified in 6 patients, classified as UICC stage IVc.

On immunohistochemistry, 25.0% were p16-positive (29.3% center 1 vs 17.4% center 2), and 3.6% were EBV-positive (3.7% center 1 vs 0% center 2). Using TNM 8, p16-positive patients were downstaged within UICC stage IV, whereas p16-negative patients were upstaged to UICC IVc. Between-center differences were not statistically significant for most baseline variables, with the exception of N classification (UICC 8; *P* = .019), histologic grading (*P* = .014), and age at initial diagnosis (*P* < .001).

### Overall survival

**Figure 1:**
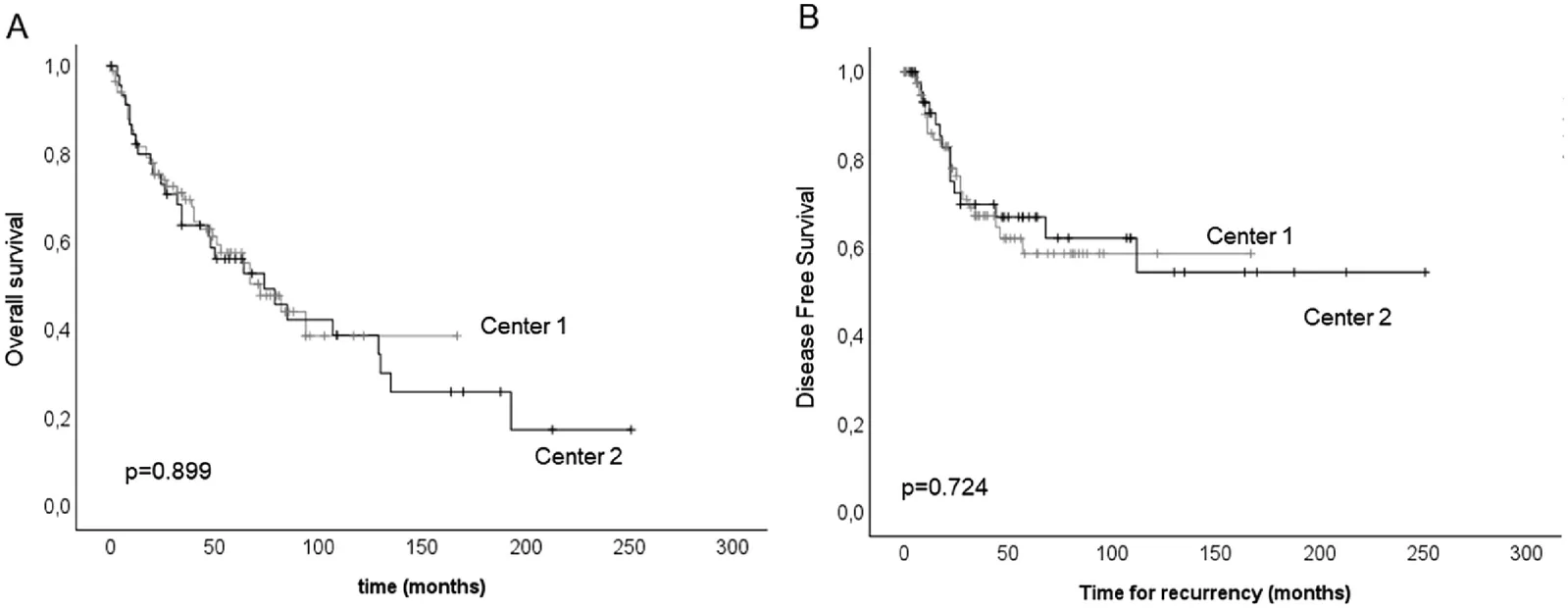
Overall survival (A) and disease-free survival (B) in patients with HNCUP. All patients underwent neck dissection with adjuvant irradiation to the lymphatic drainage or definitive chemoradiation therapy; in center 2, an additional 50 Gy pan-mucosal irradiation to the upper aerodigestive tract was delivered.

After a median follow-up of 72 months (95% CI, 48-96 months), 65 patients (42%) had died (37 in center 1 and 28 in center 2). In center 1, the 1-, 3-, and 5-year OS rates were 82.9%, 72.6%, and 57.5%, respectively. In center 2, corresponding OS rates were 82.2%, 63.7%, and 56.1%. OS did not differ significantly between centers (log-rank *P* = .899; Fig. 1A).

### Disease recurrence

Tumor recurrence occurred in 39 of the 134 patients: 24 of the 88 (27.3%) in center 1 and 15 of the 46 (32.6%) in center 2 (*P* = .519). The 1-, 3-, and 5-year DFS rates for the entire cohort were 87.8%, 68.4%, and 62.6%, respectively, with no significant difference between centers (log-rank *P* = .724; Fig. 1B).

Sites of recurrence included intracranial, cutaneous, pulmonary, axillary, and cervical lymph nodes (Table 4). Mucosal recurrences within the larynx, pharynx, or oral cavity were observed in 13 patients (9 in center 1 and 4 in center 2; *P* = .282). Notably, all mucosal recurrences occurred more than 36 months after the initial HNCUP diagnosis, and all affected patients were persistent smokers.

**Table 4:** Localizations of tumor recurrences

Localization	Total N = 90 (67.1%)	Center 1 N = 60 (68.2%)	Center 2 N = 30 (65.2%)	*P*-value = .519
Mucosal HNSCC	13 (9.7%)	9 (10.2%)	4 (8.7%)	*
Nll. cervical	16 (11.9%)	12 (13.6%)	4 (8.7%)	
Nll. axillary	2 (1.5%)	1 (1.1%)	1 (2.1%)	
Pulmo	11 (8.2%)	6 (6.8%)	5 (10.4%)	
Dermis	2 (1.5%)	0	2 (4.2%)	
Liver	2 (1.5%)	2 (2.3%)	0	
Intestine	1 (0.7%)	1 (1.1%)	0	
Intracranial	1 (0.7%)	1 (1.1%)	0	
Bone	2 (1.5%)	1 (1.1%)	1 (2.1%)	

Abbreviations: HNSCC = head and neck squamous cell carcinoma.

⁎ Fisher’s exact test comparing center 1 and 2 for mucosal HNSCC, *P* = .771.

## Discussion

HNCUP is characterized by cervical nodal metastases without identification of a primary tumor despite comprehensive diagnostic work-up, most commonly representing squamous cell carcinoma of the upper aerodigestive tract.^1^, ^2^, ^3^ Contemporary management includes ND followed by adjuvant RT or CRT or definitive RT/CRT.^18^ The role of elective irradiation of potential mucosal primary sites remains controversial and varies between institutions.^21^^,^^25^, ^26^, ^27^

In this study, we compared 2 institutional approaches: nodal irradiation without elective mucosal coverage versus the addition of pan-mucosal irradiation. Baseline characteristics were broadly comparable between cohorts, including stage distribution, viral status (HPV/p16, EBV), and exposure to tobacco and alcohol, with age being the main difference. We observed no significant differences in OS, DFS, or mucosal recurrence rates between treatment strategies. These findings suggest that routine addition of pan-mucosal irradiation may not provide a clear oncologic benefit in a thoroughly staged HNCUP population treated with modern RT techniques.

Several factors may contribute to these findings. Modern staging, including fluorodeoxyglucose-PET/CT scan, increases the likelihood of identifying occult primary tumors and may reduce the incidence of subsequent mucosal emergence.^17^^,^^18^ In addition, HPV-associated disease is known to confer a more favorable prognosis, whereas EBV positivity is primarily relevant in nasopharyngeal carcinoma,^28^ although its prognostic role in HNCUP remains uncertain.^29^, ^30^, ^31^

A key consideration in the interpretation of our results is the technique. In center 1, IMRT techniques were used throughout the study period, with a transition to volumetric modulated arc therapy over time. Although no elective mucosal target volume was defined, incidental dose to adjacent mucosal structures cannot be excluded. Therefore, the absence of differences in mucosal recurrence between centers must be interpreted with caution, particularly in the absence of detailed dosimetric data.

Our findings are consistent with previous retrospective studies showing no clear oncologic advantage of more extensive mucosal irradiation compared with nodal-only approaches.^32^ At the same time, evidence from prospective studies remains limited, and institutional practices continue to vary widely.^21^^,^^25^, ^26^, ^27^

An important observation in our cohort was the pattern of recurrence. Most failures were nonmucosal, including nodal, pulmonary, cutaneous, and intracranial sites. Mucosal recurrences were infrequent, occurred late (>36 months), and were observed only in patients with ongoing tobacco exposure. This pattern suggests that these lesions may be more likely to represent second primary tumors rather than delayed manifestations of an occult index tumor,^26^ although this cannot be definitively established in a retrospective analysis. It is also important to consider that a proportion of patients in both centers had a history of prior malignancy, which may have contributed to the observed distribution of distant and nonmucosal recurrences. This underscores the biological heterogeneity of this patient population and the complexity of attributing recurrence patterns solely to initial treatment strategy.

Beyond oncologic outcomes, treatment-related toxicity is a relevant consideration. Elective mucosal irradiation increases dose exposure to structures involved in swallowing and salivation and may therefore contribute to xerostomia and dysphagia. Prior studies have demonstrated that reducing mucosal dose—particularly with larynx-sparing approaches or advanced techniques such as IMRT and proton therapy—can significantly decrease rates of xerostomia, dysphagia, and long-term functional impairment (eg, mucositis, xerostomia).^33^^,^^34^ Therefore, in the absence of a clear survival benefit, the routine use of pan-mucosal irradiation should be carefully weighed against its potential toxicity burden.

Taken together, our results do not support a routine use of pan-mucosal irradiation in all patients with HNCUP following comprehensive staging and modern RT.^25^^,^^26^^,^^33^^,^^34^ However, given the retrospective design, potential selection bias, heterogeneity in RT techniques, and lack of detailed dosimetric data, these findings should not be interpreted as definitive evidence against mucosal irradiation. Treatment decisions should therefore be individualized based on patient- and disease-related risk factors, including smoking history, nodal distribution, and diagnostic certainty.

Future prospective studies with standardized target definitions, detailed dosimetric assessment, and incorporation of patient-reported outcomes are needed to better define the optimal RT strategy in HNCUP and to clarify whether incidental mucosal dose in modern nodal irradiation is sufficient to obviate formal pan-mucosal treatment.

## Conclusion

In this retrospective 2-center cohort of thoroughly staged patients with HNCUP, the addition of elective pan-mucosal irradiation to standard nodal irradiation was not associated with improved survival or reduced mucosal recurrence compared with nodal-only treatment. Given the potential for increased toxicity and quality-of-life impact, routine use of pan-mucosal irradiation may not be warranted. Treatment volume selection should be individualized, and prospective studies are needed to further refine optimal RT strategies in this patient population.

## Disclosures

None.
